# P267: Current aspects of acquired infection in maternity Issaka Gazobi hospital – Niamey, Niger

**DOI:** 10.1186/2047-2994-2-S1-P267

**Published:** 2013-06-20

**Authors:** H Djibo, M Kamay, A Baden, N Madi, KM Issaka, M Garba, S Alassane

**Affiliations:** 1 Department of Public Health, Faculty of Health Sciences, University of Niamey, Niamey, Niger; 2 Health Sector Control Unit against STI / HIV / AIDS , Ministry of Public Health, Niamey, Niger; 3 Niamey National Hospital, Niamey, Niger

## Objectives

This document is the result of a study of 1 November 2010 to 30 March 2011 on nosocomial infections at the Maternity Issaka Gazobi.

## Methods

This prospective survey covered a total of 139 patients of Obstetrics.

## Results

After analyzing the data, it appears that: The prevalence of nosocomial infections was 7.2%, including 0.7% of endometritis, urinary tract infection 0.7% and 5.8% of surgical site infection distributed as follows: 3.5% and 2.2% of deep and superficial infections respectively. Nearly eight out of 10 patients were from the urban community of Niamey and 70% of infected patients are housewives. Prolonged urinary catheterization, as well as antibiotic misuse were the main contributing factors. In newborns, the prevalence of nosocomial infections was 5% of which 1.4% were skin infections and 3.6% eye infections, no umbilical cord infection was recorded. The mortality rate of newborns was 14.4%. Nosocomial infections are and remain a public health problem.

## Conclusion

This is what we contribute to formulate recommendations for the administrative and health authorities and for healthcare workers.

## Competing interests

None declared

